# Evaluation of White Blood Cell Count, Lymphocyte Percentage, Neutrophil Percentage, and Elevated Temperature as Predictors of Wound Infection in Burn Patients

**DOI:** 10.7759/cureus.63172

**Published:** 2024-06-26

**Authors:** Ali AlHawaj, Maryam AlMadhoob, Reem Alkhanaizi, Ahmed AlHaddad

**Affiliations:** 1 Plastic Surgery, King Fahad Specialist Hospital, Dammam, SAU; 2 Plastic Surgery, Queen Elizabeth Hospital Birmingham, Birmingham, GBR; 3 Medicine, Salmaniya Medical Complex, Manama, BHR; 4 Plastic Surgery, Royal College of Surgeons in Ireland - Medical University of Bahrain, Muharraq, BHR; 5 Plastic Surgery, Salmaniya Medical Complex, Manama, BHR

**Keywords:** high temperature, neutrophil percentage, lymphocyte percentage, white blood count (wbc), burn wound infection, burn wounds

## Abstract

Introduction: Infection remains a chief cause of morbidity and mortality among burn patients. The burn wound surface is initially sterile after a thermal injury but eventually gets colonized by microorganisms. A burn wound is considered infected upon the presence of high concentrations of microorganisms in the wound and scab.

Burn wound infections can lead to a delay in epidermal maturation, higher scar formation, and sepsis. However, burn patients are commonly misclassified as septic due to the manifestation of systemic inflammatory response syndrome (SIRS) after their injury, despite the presence or absence of an infection.

Methods: This is a retrospective review of medical records of patients admitted to the burn unit in Salmaniya Medical Complex in Manama, Bahrain, between the years 2018 and 2020. Demographic data, total body surface area (TBSA), initial temperature, white blood cell count, lymphocyte percentage, neutrophil percentage, and wound cultures were obtained for all subjects. Logistic regression analysis was performed to compare the presence or absence of wound infection by the aforementioned parameters.

Results: Of 412 cases, 68.2% were male patients, with a mean age for the studied population of 25.1 years (standard deviation (SD)=20.7). Staphylococcus aureus was the most prevalent organism across all of the study population (n=31)(34.4%). Staphylococcus aureus was the most prevalent organism in patients under the age of five, while Pseudomonas aeruginosa was the most common organism among adults older than 65 years of age. TBSA was not found to be a good predictor of wound infection. There was no statistically significant relation between initial temperature and wound culture (p-value=0.056). However, logistic regression revealed that the initial temperature increases the likelihood of positive wound culture by almost three times.

Conclusion: White blood cell count, lymphocyte percentage, and neutrophil percentage were not clinically reliable in predicting burn wound infection. However, initial temperature might be a helpful predictor. Further research is needed to identify reliable clinical parameters of burn wound infections.

## Introduction

Infection remains a chief cause of morbidity and mortality among burn patients [1]. The burn wound surface is initially sterile after a thermal injury but eventually gets colonized by microorganisms [2,3]. A burn wound is considered infected upon the presence of high concentrations of microorganisms (>10^5^ organisms/g of tissue) in the wound and scab [4].

Burn wound infections can lead to a delay in epidermal maturation, higher scar formation [5,6], and sepsis [7]. However, burn patients are commonly misclassified as septic due to the manifestation of systemic inflammatory response syndrome (SIRS) after their injury, despite the presence or absence of an infection [8,9].

Burn injuries have a major effect on the immune system [10,11]. A pro-inflammatory state is the initial response to severe burn injuries, followed by an anti-inflammatory phase to restore homeostasis [12]. Burn injuries can compromise many different immune functions including the suppression of T-cell proliferation and IL-2 production. Additionally, antigen presentation by macrophages and the killing of invading pathogens by neutrophils can also be severely compromised, making the patient susceptible to infectious complications [13].

White blood cell counts of more than 12,000 cells/mm^3^ and less than 4,000 cells/mm^3^ are frequently used as laboratory indicators for the possible presence of an infection. Changes in body temperature are another cardinal indicator of infections. The original SIRS definition includes temperatures of more than 38°C and less than 36°C [14].

Regular monitoring of vital signs and careful inspection of the burn wound surface with each dressing change are the key principles for a clinical diagnosis of an infected burn wound [12]. In this research, we aimed to evaluate the use of white blood cell count, lymphocyte percentage, neutrophil percentage, and elevated temperature as predictors for the presence of wound infection in burn patients.

## Materials and methods

This is a retrospective study. The inclusion criterion in this paper is all of the medical records for patients admitted to the Burn Unit in Salmaniya Medical Complex, Manama, Bahrain, between 2018 and 2020. The exclusion criterion is any patients with missing data. 

Patients' data included in this study are age, sex, total body surface area (TBSA) burned, wound culture, and initial temperature. Additionally, white blood cells, lymphocytes, and neutrophils count, as well as absolute neutrophil count, were included. These data were collected and investigated to predict the likelihood of wound infection. Moreover, the initial temperature was recorded for all participants and classified as normal (36.5-37.5°C), high (>37.5°C), and low (<36.5°C).

Descriptive analysis was used to present the findings, which include tables, charts, mean, and standard deviation. Also, in order to demonstrate the distribution and skewness of the results as well as detect outliers, a box plot was used. The inferential level statistical analysis included a normality test, and a bivariate test for any differences due to demographic variables. Non-parametric test Mann-Whitney was used as the bivariate test and statistical significance was set at p<0.05. Ethical approval was obtained from the Secondary Health Care Research Committee of the Kingdom of Bahrain.

## Results

A total of 412 cases were reviewed in this study. The majority of the cases were male patients (n=281) (68.2%). The mean age for the studied population was 25.1 years (standard deviation (SD)=20.7). Data collected in this study included patients' age, TBSA, initial temperature, white blood cell count, lymphocyte percentage, and neutrophil percentage and count (Table 1). 

**Table 1:** Summary of the collected data.

Parameter	No. of patients		Mean	Median	Standard deviation	Minimum	Maximum
	Valid	Missing					
Age (years)	412	0	25.0878	29.0000	20.68314	.05	85.00
Burn %	368	44	11.268	8.000	12.5836	1.0	90.0
Initial temperature	403	9	36.929	37.000	.3435	35.0	39.4
WBC	308	104	11.227	10.200	5.2949	2.5	34.1
Lymphocyte %	308	104	31.173	27.400	18.3828	4.3	88.9
Neutrophil %	308	104	60.441	62.750	19.0536	7.3	91.6
Neutrophil count	307	105	7.0047	6.0000	4.58290	.45	28.00

After excluding cases with missing data for wound cultures (n=46), most of the cases had negative wound cultures (n=292) (79.8%). Among the cases with a positive wound culture (n=74)(20.2%), Staphylococcus aureus was the most prevalent organism to be isolated (n=31) (34.4%) (Table 2).

**Table 2:** The prevalence of organisms isolated from positive wound cultures.

Organism	No. of patients	%
Staphylococcus aureus	31	34.4
methicillin-resistant Staphylococcus aureus (MRSA)	12	13.3
Escherichia species	6	6.7
Enterobacter species	7	7.8
Acinetobacter species	8	8.9
Klebsiella species	4	4.4
Pseudomonas aeruginosa	7	7.8
Candida species	2	2.2
Streptococci	6	6.7
Other organisms	7	7.8
Total	90	100.0

Staphylococcus aureus was also the most common organism to be isolated in patients under the age of five, while Pseudomonas aeruginosa was the most common organism found in positive wound cultures of patients at 65 years of age or older (Table 3).

**Table 3:** Organism distribution by age category. N: no. of patients

Organism	Age category					Total
	Less than five years		65 years or above			
	N	%	N	%	N	
Staphylococcus aureus	9	81.8%	2	18.2%	11	
Methicillin-resistant Staphylococcus aureus (MRSA)	2	66.7%	1	33.3%	3	
Escherichia species	4	80.0%	1	20.0%	5	
Enterobacter species	1	100.0%	0	0.0%	1	
Acinetobacter species	4	100.0%	0	0.0%	4	
Klebsiella species	2	100.0%	0	0.0%	2	
Pseudomonas aeruginosa	0	0.0%	3	100.0%	3	
Candida species	1	100.0%	0	0.0%	1	
Streptococci	2	66.7%	1	33.3%	3	
Other organisms	3	100.0%	0	0.0%	3	
Total	28		8		36	

Moreover, normality tests showed that the variables (patients' age, TBSA, initial temperature, white blood cell count, lymphocyte percentage, and neutrophil percentage and count) in this study were not normally distributed. Hence, the Mann-Whitney non-parametric test was employed as it does not assume normality. Mann-Whitney test showed that there was only one statistically significant difference, which was the TBSA in relation to wound culture (Z = -2.767, p-value = 0.006) at a 1% significance level (Table 4).

**Table 4:** Mann-Whitney analysis of clinical parameters. ^a ^grouping variable: wound culture

Parameter	Mann-Whitney test^a^			
	Mann-Whitney U	Wilcoxon W	Z	p-value
Age (years)	9827.000	52605.000	-1.202	.229
Burn %	6447.500	8338.500	-2.767	.006
Initial temperature	9777.500	51682.500	-1.016	.310
WBC	6155.500	8300.500	-1.800	.072
Lymphocyte %	6596.500	8741.500	-1.051	.293
Neutrophil %	6641.500	31394.500	-.975	.330
Neutrophil count	6744.000	8889.000	-.751	.452

However, receiver operating characteristic curve analysis determined TBSA to be a poor discriminator. Thus, TBSA cannot be used as a predictor of wound infection. Moreover, the chi-square test showed no statistically significant relation between initial temperature and wound culture (p-value = 0.056). However, logistic regression revealed that the initial temperature increases the likelihood of positive wound culture by almost three times (Table 5).

**Table 5:** Logistic regression of burn percentage and initial temperature as predictors of wound culture result.

Predictor	Dependent variable: wound culture (negative = 0 vs. positive = 1)	
	Odds ratio	p-value
Burn %	0.962	.025
Initial temperature	2.787	.021
Constant	0.000	.031

## Discussion

In this study, most patients were males. This is consistent with the demographic pattern of burn populations in recent epidemiological studies [15,16]. Moreover, Staphylococcus aureus was the most common infective organism isolated from burn wound cultures. This is similar to the results of a recent epidemiology study done in the largest burn unit in Saudi Arabia [16].

In this study, TBSA in relation to wound culture was the only statistically significant result found, where negative wound cultures had higher TBSA. This is inconsistent with the results of a recent Saudi study [16], which found that 100% of pediatric burns had a positive blood and wound culture when TBSA was more or equal to 40%. This Saudi study concluded that TBSA could not be used as a predictor for a positive wound culture result [16]. Additionally, another study revealed that the TBSA of a burn wound increases the proportion of microbial isolates. It also found that the extent of TBSA is the most important factor affecting multiple drug-resistant organisms [17]. Also, a study from Lebanon found that infected patients had significantly higher TBSA burns when compared to non-infected burn patients and were more likely to present with third-degree burns [18]. 

All other parameters (patients' age, initial temperature, white blood cell count, lymphocyte percentage, and neutrophil percentage and count) were not found to be predictive of positive wound infections. Similar results were found in a study researching the relationship between these parameters and bloodstream infections in burn patients [19]. 

Although this paper aims to find a relation between the aforementioned parameters and the presence of burn wound infections. It is important to take into consideration the difference between an infection and the colonization of a burn wound, as both can give positive wound culture results. Hence, it is important to keep other clinical signs in mind like the presence of purulent discharge, erythema, swelling, and pain when assessing any patient with a burn wound [20]. Wound surveillance with regular sampling of tissues for quantitative culture, early excision, and wound closure remain the main principles to control invasive infections in burn patients [4].

Lastly, this study is the first of its kind in Bahrain to identify the demographics of the burn population. We now have a baseline of burn infection trends in the region. This study also supports the results of the previous studies that attempted to find reliable parameters for predicting infections in burn patients. However, this study is based on a relatively small population as it only includes patients from one burn center in Bahrain. Moreover, there are discrepancies in the collected data as some patients had their initial data collected in the emergency room and some had their initial data collected inside the burn unit.

## Conclusions

This study investigated different parameters (TBSA, initial temperature, white blood cell count, lymphocyte percentage and neutrophil percentage) as predictors of positive burn wound culture results. None of the investigated parameters were found to be sufficient enough to predict a positive culture result. Hence, we conclude that further research is needed to identify more reliable clinical parameters for burn wound infections. 
